# Impact of the COVID-19 pandemic on the clinical training of last year medical students in Mexico: a cross-sectional nationwide study

**DOI:** 10.1186/s12909-021-03085-w

**Published:** 2022-01-08

**Authors:** Maximiliano Servin-Rojas, Antonio Olivas-Martinez, Michelle Dithurbide-Hernandez, Julio Chavez-Vela, Vera L. Petricevich, Ignacio García-Juárez, Alice Gallo de Moraes, Benjamin Zendejas

**Affiliations:** 1 Faculty of Medicine, Universidad Autónoma del Estado de Morelos, Cuernavaca, Morelos Mexico; 2 Department of Biostatistics, University of Washington, Seattle, Washington USA; 3 Liver Transplant Unit, Instituto Nacional de Ciencias Médicas Y Nutrición “Salvador Zubirán”, Mexico City, Mexico; 4 Division of Pulmonary and Critical Care Medicine, Mayo Clinic, Rochester, Minnesota USA; 5 Department of Surgery, Boston Children Hospital, Harvard Medical School, 300 Longwood Avenue, Boston, MA 02115 USA

**Keywords:** COVID-19, Medical education, Mexico, Medical students

## Abstract

**Background:**

The COVID-19 pandemic has brought unprecedented changes to medical education. However, no data are available regarding the impact the pandemic may have on medical training in Mexico. The aim of our study was to evaluate and identify the medical school students’ perceptions of the changes in their clinical training due to the pandemic in Mexico.

**Methods:**

This was a cross-sectional study where a previous validated online survey was translated and adapted by medical education experts and applied to senior medical students from March to April of 2021. The 16-item questionnaire was distributed online combining dichotomous, multiple-choice, and 5-point Likert response scale questions. Descriptive and multivariate analyses were performed to compare the student’s perceptions between public and private schools.

**Results:**

A total of 671 responses were included in the study period. Most participants were from public schools (81%) and female (61%). Almost every respondent (94%) indicated it was necessary to obtain COVID-19 education, yet only half (54%) received such training. Students in private schools were less likely to have their clinical instruction canceled (53% *vs.* 77%, *p* = 0.001) and more likely to have access to virtual instruction (46% *vs.* 22%, *p* = 0.001) when compared to students from public schools. Four out of every five students considered their training inferior to that of previous generations, and most students (82%) would consider repeating their final year of clinical training.

**Conclusions:**

The impact of the COVID-19 on medical education in Mexico has been significant. Most final-year medical students have been affected by the cancellation of their in-person clinical instruction, for which the majority would consider repeating their final year of training. Efforts to counterbalance this lack of clinical experience with virtual or simulation instruction are needed.

## Background

The COVID-19 pandemic has changed the education of health professions [1]. In March 2020, the General Health Council of Mexico declared COVID-19 a national health emergency and took measures to mitigate the burden of the disease [2]. The Mexican government suspended all public and private activities that were considered non-essential. Most medical schools decided to cancel in-person teaching and moved to virtual or remote education. The Association of American Medical Colleges (AAMC) and its Mexican equivalent, the Asociacion Mexicana de Facultades y Escuelas de Medicina (AMFEM) recommended suspending all clinical activities [1]. Medical schools in Mexico aligned with these recommendations and removed medical students from hospital or outpatient-based settings [3].

Exposure to the clinical environment is a vital part of the training of a physician [4]. Through observation, practice and repetition, usually under the guidance of expert clinicians, medical students learn essential clinical skills to care for patients. Though some of these clinical skills can be taught in a non-clinical setting, with the help of simulation-based instruction, traditional bedside training remains a cornerstone of medical education [5]. The COVID-19 mitigation measures implemented in Mexico and other parts of the globe might significantly hinder the clinical skills training of medical students. This could have deleterious effects on their confidence and capability to care for patients and their autonomy as physicians. Additionally, studies have shown higher rates of mental health disorders during the pandemic [6, 7]. With campus and hospital-based teaching suspended, medical students are unable to participate in bedside training. Furthermore, simulation-training is often done in group settings or on-campus, for which the use of simulation, as an alternative to clinical training during the COVID pandemic, is challenging to implement given the social distancing guidelines that limit group gatherings and on-campus instruction.

The impact of these issues on the training of current medical students is unknown. Studies conducted in different parts of the world have shown that an important proportion of medical students feel less prepared due to these public health restrictions and changes to their clinical education [8–10]. In some cases, medical students were willing to participate in clinical instruction even if there was a risk for infection [10]. In low-income countries, there also exists a significant challenge to design and implement effective remote clinical education strategies, where a substantial proportion of the students consider remote strategies inadequate [9, 11]. Furthermore, heterogeneity among Mexican medical schools programs exists, as only few medical schools have alternatives to bedside training, and these issues might affect students unequally [12].

In order to better understand the impact of the COVID-19 pandemic on the training of medical students in Mexico, we surveyed the opinions of medical students in their last year of training. We hypothesized that most medical students in Mexico would feel unprepared to graduate from medical school and that many would choose to either extend their training or repeat their final year of medical school once clinical instruction could resume. The aim of our study was to evaluate and identify the medical school students’ perceptions of the changes in their clinical training due to the pandemic in Mexico.

## Methods

This was an exploratory, observational, and cross-sectional study. An online survey was distributed and conducted from March to April of 2021. The 16-item questionnaire was distributed online via the Google Forms platform (Alphabet, Mountain View, CA, USA). With the endorsement of the Asociación Mexicana de Médicos en Formación (AMMEF), a medical student association involved in medical education activities, the survey was distributed among social media platforms (Facebook™ and Twitter™), and via email to their last year medical student affiliates. The questionnaire was self-administered. We defined last year medical students as those in their final year of clinical (before entering hospital-based undergraduate internship). Students in the last year of medical school face a transition period between medical school and full-time patient care in the hospital-based scenario in the internship year. Virtual rotations were defined as observation of clinical practice via telemedicine under the supervision of an attending physician. Medical schools were categorized by type of institution (public *vs.* private) and were grouped into 4 different regions according to their geographical location: Northern, Central, and Southern Mexico, and Mexico City. Only participants in their last year of medical school who consented to participate were included. Exclusion criteria included medical students who did not provide consent or were not in their last year of medical education.

### Survey Tool

We evaluated medical student’s perceptions using a previously validated questionnaire [8]. The survey was translated from English into Spanish by a bilingual expert in medical education and some extra questions that applied to the uniqueness of medical training in Mexico were developed. Questions were iteratively reviewed and revised by all study members. Before its implementation, the expert further revised the appropriateness of the questionnaire. Additionally, during the survey distribution, an email was provided for students to provide feedback. The final questionnaire comprised of 16 items, combining dichotomous, multiple choice and 5-point Likert response scale questions. The survey can be accessed with the following link: https://docs.google.com/forms/d/e/1FAIpQLSf9p9kZFsA4vpK7Z0dLh8fcIENshNlDb4eAkUc1lSNe9N6blw/viewform?usp=sf_link

### Sample size

Sample size was calculated based on the latest report of the Mexican Health Ministry [13]*,* in which 22,160 last year medical students were eligible for this study*.* To obtain the sample size, and considering a value of α = 0.05 and a value of 1- β of 0.80, we used the following formula:

$$n = [\mathrm{EDFF}+\mathrm{Np}(1-\mathrm{p})]$$

$$[(\mathrm{d}2/\mathrm{Z}2 1-\mathrm{ \alpha }/2*(\mathrm{N}-1)+\mathrm{p}*(1-\mathrm{p})$$

The above power calculation resulted in a sample size of 645 students with a CI of 99%.

### Statistical Analysis

Descriptive statistics are presented as median and interquartile range (IQR) for numerical variables and frequencies and percentages for categorical variables. Categorical variables were compared using Pearson’s Chi Squared Test or Fisher’s Exact Test as appropriate. The level of statistical significance was set as *p* < 0.05. To evaluate if medical student perceptions regarding clinical training differ by the type of institution (private versus public), we transformed each item into a binary variable and for each of these binary items we fitted a multivariate logistic regression model, using robust standard error estimates, including the item as outcome, the type of institution as a predictor (1 = private, 0 = public) and adjusting age, gender, and geographical region. All statistical analyses were performed using R v.4.0.5. (R Foundation for Statistical Computing, Vienna, Austria. URL http://www.R-project.org/).

## Results

A total of 721 survey responses were obtained during the study period, representing 3% of the eligible medical student population. Fifty surveys were excluded since 7 students did not consent to participate in the study and 43 were from students not enrolled in their last year of medical school. Therefore, 671 surveys were finally included and analyzed.

### Demographic Variables

Most of the participants studied in public schools (81%). Overall, 61% of the participants were female with a median age of 22 years (IQR 22—23). The majority of the students were enrolled in schools from Central Mexico (35%), followed by Northern (30%) and Southern (24%) regions, while only 11% where from schools in Mexico City (11%) (Table 1). The comparisons of participants’ demographics characteristics between groups are summarized in Table 1.

**Table 1:** Demographics of survey respondents

	Total (*n* = 671)	Private (*n* = 129)	Public (*n* = 542)
	n (%)	n (%)	n (%)
**Age (years)**^**a**^	22 (22, 23)	22 (22, 2)	22 (22, 23)
**Sex**			
Male	263 (39)	32 (25)	231 (43)
Female	408 (61)	97 (75)	311 (57)
**Region**			
Mexico City	73 (11)	11 (8.5)	62 (11)
Central Mexico	238 (35)	57 (44)	181 (33)
Northern Mexico	202 (30)	22 (17)	180 (33)
Southern Mexico	158 (24)	39 (30)	119 (22)

^a^Median (IQR)

### Education on COVID-19

Despite almost every participant (97%) stated they “Agreed” or “Completely Agreed” that it was necessary to receive COVID-19 education, only around half of the students (54%) reported having received COVID-19 related education from their medical schools. Overall, the majority of the COVID-19 related medical education provided was in the form of in-person or virtual lectures (57%), followed by educational pamphlets or handouts (15%). Only 11 students reported simulation as a source of COVID-19 education. No statistical differences were found regarding COVID-19 education between groups (Table 2).

**Table 2:** Medical student perceptions regarding COVID-19 education

	Total ( *n* = 671)	Private (*n* = 129)	Public (*n* = 542)
	n (%)	n (%)	n (%)
**Have you received COVID-19 education?**			
Yes	364 (54)	71 (55)	293 (54)
No	307 (46)	58 (45)	249 (46)
**Do you consider necessary receiving COVID-19 education?**			
Completely Agree	575 (86)	105 (81)	470 (87)
Agree	77 (11)	19 (15)	58 (11)
Neutral	18 (2.7)	5 (3.9)	13 (2.4)
Disagree	1 (0.1)	0 (0)	1 (0.2)
Completely Disagree	0 (0)	0 (0)	0 (0)
**What kind of COVID-19 education have you received?**			
In Class or Virtual Lecture	385 (57)	77 (60)	308 (57)
Educational Pamphlet	101 (15)	17 (13)	84 (15)
Simulation	11 (1.6)	6 (4.7)	5 (0.9)
Social Media	2 (0.3)	0 (0)	2 (0.4)
None	129 (19.7)	27 (20.7)	102 (19.2)
Other	3 (0.4)	0 (0)	3 (0.6)
No response	40 (6.0)	2 (1.6)	38 (7.0)

### Perceptions Regarding Clinical Training

Most students considered clinical rotations as “Very Important” (94%). When compared between groups, similar rates were found (private 95% *vs.* public 94%; *p* = 0.7). Students from private schools were less likely to have their rotations cancelled (53% *vs.* 77%; *p* = 0.001) and were more likely to have virtual rotations, (46% *vs.* 22%; *p* = 0.001). The majority of the participants considered their medical training was affected by the COVID-19 (private 95% *vs.* 96% public; *p* = 0.65). Also, most students agreed these changes would negatively impact their performance as hospital-based interns (private 99% *vs.* 96% public; *p* = 0.10). Interestingly, a greater proportion of public-school students “Completely agreed” or “Agreed” that restrictions were necessary compared with students from private schools (78% *vs.* 60%, *p* = 0.001). Moreover, a lower proportion of the public-school students (37%) indicated they would feel safe going back to their clinical rotations. Four out of every five students (79% and 82% in public and private schools, respectively) felt that their clinical training was worse than their peers from previous generations not affected by the COVID-19 restrictions. A higher proportion of participants indicated that, if possible, they would repeat the final year of medical school training (81% public *vs.* 85% private; *p* = 0.3). Further details about medical students’ perceptions about their clinical training are provided in Table 3.

**Table 3:** Medical student perceptions regarding clinical training

		Total ( *n* = 671)		Private (*n* = 129)		Public (*n* = 542)	*p*-value*
		n (%)		n (%)		n (%)	
**How important do you consider clinical rotations in your training?**							0.7
Very Important		632 (94)		122 (95)		510 (94)	
Important		34 (5.1)		6 (4.7)		28 (5.2)	
Moderately Important		2 (0.3)		1 (0.8)		1 (0.2)	
Slightly Important		2 (0.3)		0 (0)		2 (0.4)	
Not Important		1 (0.1)		0 (0)		1 (0.2)	
**What changes have you experienced in your clinical rotations?**							** < 0.001**
Cancelled		486 (72.5)		68 (52.7)		418 (77.1)	
Virtual Rotations		182 (27.1)		60 (46.5)		122 (22.5)	
No changes		3 (0.4)		1 (0.8)		2 (0.4)	
**How do you think these changes will affect your medical training?**							0.065
Positively		12 (1.8)		5 (3.9)		7 (1.3)	
Neutral		16 (2.4)		1 (0.8)		15 (2.8)	
Negatively		643 (96)		123 (95)		520 (96)	
**Do you consider these changes will affect your performance as pregrade intern?**							0.10
Yes		649 (97)		128 (99)		521 (96)	
No		22 (3.3)		1 (0.8)		21 (3.9)	
**Do you consider the restrictions necessary?**							**0.001**
Completely Agree		221 (33)		33 (26)		188 (35)	
Agree		279 (42)		44 (34)		235 (43)	
Neutral		116 (17)		35 (27)		81 (15)	
Disagree		45 (6.7)		14 (11)		31 (5.7)	
Completely Disagree		10 (1.5)		3 (2.3)		7 (1.3)	
**Would you feel safe going back to your clinical rotations?**							** < 0.001**
Yes	276 (41)		76 (59)		200 (37)		
No	395 (59)		53 (41)		342 (63)		
**In comparison to previous generations not affected by the pandemic, your feel that your clinical training was**							0.10
Better	85 (13)		19 (15)		66 (12)		
Equal	50 (7.5)		4 (3.1)		46 (8.5)		
Worse	536 (80)		106 (82)		430 (79)		
**If it was possible to do so, would you repeat final year training?**							0.3
Yes	551 (82)		110 (85)		441 (81)		
No	120 (18)		19 (15)		101 (19)		

^*^Fisher's exact test; Pearson's Chi-squared test

### Multivariate Logistic Regression Analysis

Among subjects of the same age, gender, and geographical region, we estimated that, when comparing subjects who attend private and public institutions, the odds ratio for having virtual rotations was 1.99 (95% CI 1.30 – 3.04, *p* < 0.001), the OR of agreeing that restrictions were necessary was 0.41 (95% CI 0.27 – 0.62, *p* < 0.001), and the OR of feeling safe going back to the clinical rotations was 0.39 (95% CI 0.26 – 0.58, *p* < 0.001). The full results of the multivariate logisitic regression analysis can be found in Table 4.

**Table 4:** Multivariate logistic regression analysis of medical student perceptions regarding clinical training

		Private, *n* = 129	Public, *n* = 542	aOR^*^, 95% CI		*p*-value^*^
		n(%)	n(%)			
**How important do you consider clinical rotations in your training?**						
Important		128 (99)	538 (99)	0.97, 0.10 – 9.86		0.98
Not Important		1 (1)	4 (1)			
**What changes have you experienced in your clinical rotations?**						
Virtual Rotations		60 (47)	122 (23)	1.99, 1.30 – 3.04		< 0.001
Cancelled		69 (53)	420 (77)			
**How do you think these changes will affect your medical training?**						
Positively		6 (5)	22 (4)	0.76, 0.28 – 2.05		0.59
Negatively		123 (95)	520 (96)			
**Do you consider these changes will affect your performance as pregrade intern?**						
Yes		128 (99)	521 (96)	4.83, 0.67 – 35.0		0.12
No		1 (1)	21 (4)			
**Do you consider the restrictions necessary?**						
Agree		77 (60)	423 (78)	0.41, 0.27 – 0.62		< 0.001
Disagree		52 (40)	119 (22)			
**Would you feel safe going back to your clinical rotations?**						
Yes	76 (59)		200 (37)		0.39, 0.26 – 0.58	< 0.001
No	53 (41)		342 (63)			
**In comparison to previous generations not affected by the pandemic, your feel that your clinical training was**						
Worse	106 (82)		430 (79)		1.33, 0.79 – 2.25	0.29
Equal or better	23 (18)		112 (21)			
**If it was possible to do so, would you repeat final year training?**						
Yes	110 (85)		441 (81)		1.09, 0.63 – 1.89	0.76
No	19 (15)		101 (19)			

^*^Obtained using a logistic regression model adjusting for age, sex, and region. aOR (adjusted odds ratio), considering public institutions as reference group

## Discussion

This is the first study in Mexico to evaluate last year medical student’s perceptions of changes in their clinical training during the COVID-19 pandemic and reveals several interesting observations, highlights inequities in training, and provides impetus to improve our educational environment. Students feel less prepared because of the COVID-19 pandemic restrictions on their education. In the UK, over 50% of the students felt less prepared to start postgraduate training [14], while in the US 18% of the third year medical students were willing to extend their training for an extra year [10]. These results contrast with ours, where 82% of the students were willing to extend their training. This suggests potentially a greater impact of COVID-19 in our country’s medical education system.

Medical students want formal training on COVID-19, yet very few students reported having received such formal training. This can be accomplished via remote or virtual instruction. Most medical schools in both public and private sectors opted to use traditional teaching methods such as lectures and written educational materials. Students reported very limited use of simulation technologies, which might be related to the limited access to these centers in our country [15].

The role of medical students in the COVID-19 pandemic has been controversial. Some authors consider that the involvement of medical students can be beneficial for healthcare systems, patients, and their personal development as physicians, but given the existing risk of infection, their participation should be exclusively voluntary [16]. Others agree that medical students should not be involved in any clinical activity because they are not yet fully trained clinicians, do not receive a salary and may represent a risk for the people they live with [17]. In Mexico, most public hospitals care for infected COVID-19 patients. Public hospitals have also faced a shortage of personal protective equipment and high rates of infection among healthcare workers, which makes clinical rotations a very high-risk activity for medical students [18]. Probably related to these findings, most medical students in our survey reported that they would not feel safe going back to their clinical activities.

Despite clinical rotations being regarded as very important by students, most medical schools cancelled them, and only a limited number of students received an alternative such as virtual rotations. The lack of alternatives was striking as most students considered the absence of rotations would negatively impact their training and performance in the pre-grade internship. Probably related to the perception of poor training, most students would consider repeating the last year of medical school. This highlights the importance of innovation in medical education. Some experts in medical education are proposing different teaching alternatives with the development of multimodal training strategies [19–21]. Schools can offer in-person clinical rotations when the public health recommendations of social distancing can be safely achieved and when the risk of infection remains low [19]. If this is not an option, developing a virtual curriculum can be a safe and effective alternative. Even though the physical examination is not possible during virtual rotations, they can be asked to observe and evaluate different maneuvers elicited by the attending [19]. To develop skills in interviewing, students can take history and physical examination by telemedicine from consenting patients. From these experiences, they can be expected to develop written reports that can be presented to attendings and peers for additional feedback [21]. However, significant challenges also exist in the implementation of these strategies as many countries do not have the existent infrastructure to adopt a robust virtual curriculum [9]. The cancelation of elective procedures and other procedural activities could also limit the potential exposure of the students in virtual rotations. Similarly, clinicians on the front-line are also very taxed by the extra workload of caring for patients with COVID-19 and may not have the time to participate in remote medical student instruction.

In Mexico, the Consejo Mexicano para la Acreditación de la Educación Médica (COMAEM), oversees the evaluation of the quality of medical training. Only 80 out of more than 140 medical schools are certified by this organism, suggesting that there could be unequal quality of training due to differences in the regulatory bodies that accredit each medical school [22]. This study provides further insights into the inequities in medical education in Mexico. Medical students in private schools were more likely to have virtual instruction and were less likely to have their clinical electives cancelled. These issues can likely further accentuate the gap between medical students trained in private versus public schools. Further research should explore ways to enhance medical school training opportunities for public schools.

Simulation remains another unexplored area in medical education in Mexico. Studies have shown the effectiveness of simulation in facilitating teamwork, teaching basic science, clinical and procedural skills in different scenarios [23]. Despite its proven benefits, these technologies might be difficult to implement during a global pandemic [24]. Strategies limiting the number of instructors and medical students along with the proper following of public health measures could make simulation centers relatively safe [24]. We consider simulation should be increasingly adopted by medical schools in Mexico to offer more evidence-based learning techniques for trainees. Similarly, personal at-home simulators with or without virtual feedback have been successfully used as an alternative modality to in-person simulation instruction for certain skill sets; [25, 26] and online simulation is another promising alternative under evaluation [27]. Teaching faculty is able to develop simulated medical records that students can easily access anytime. For inpatients, students can give follow up to their simulated patients and solve the different complications that might arise from admission to discharge. Even though students prefer traditional in person clinical activities, most of them appear to be satisfied with this type of training [27]. This type of curriculum could be an attractive alternative for low-income countries. It has the benefit of being low cost, [28–30] less time intensive for students and it can provide ample feedback from expert clinicians.

These results should encourage policymakers to update the Mexican regulations (Norma Oficial Mexicana, “NOM”) on medical education. Studies have reported heterogeneity among teaching hospitals [31]. We propose that teaching hospitals should undergo continued evaluations to establish quality standards [32]. Furthermore, telemedicine remains underutilized in Mexico [33]. Investing in telemedicine could improve access to healthcare in rural communities and offer learning opportunities to medical students. Medical schools should consider integrating telemedicine and simulation into their curriculum and train educators on the usage of these technologies [34]. In addition, vaccinating and training medical students on the proper use of personal protective equipment could facilitate a safe return to clinical rotations [35]. Lastly, medical schools should train faculty members to provide educational and emotional support to improve academic achievements, and most importantly, their sense of security [36].

### Limitations

This study has some limitations. Due to the study design, we can only provide a representation of the perception of last year medical students during a specific time period. The participation in this study response rate is low and might not represent the perceptions of the entire population of last year medical students in Mexico, yet it still the largest study of its kind with a significant number of survey responses of students that represent diverse geographic regions and types of medical training (public *vs.* private schools). Because our study was distributed online by social media it might be susceptible to non-response and participation bias; students with access to internet could more readily participate. Furthermore, not all medical students engage in social media platforms and hence might have been unaware of the study. We attempted to overcome this by emailing medical students, but this method has its own pitfalls, and we did not have access to the emails of all eligible medical students.

## Conclusion

The COVID-19 pandemic has impacted medical education. Medical students had to abandon clinical activities due to the inherent risks of infection. Medical schools in Mexico were unprepared to implement a rapid and effective response for this unprecedented pandemic. Medical education in Mexico is highly heterogeneous, and most medical schools have no alternatives to traditional bedside training. This has left medical students feeling unprepared for the next stages of their careers. Urgent measures must be taken to guarantee effective clinical training in situations like this and enable them to safely care for patients. Multiple alternative educational strategies exist that may be adaptable to middle and low-income countries. Medical schools should develop contingency plans to deliver effective clinical instruction that does not rely on in-person presence, in order to be prepared for future pandemics.

## Data Availability

The datasets used and/or analyzed during the current study are available from the corresponding author on reasonable request.

## Abbreviations

AAMC: Association of American Medical Colleges
AMFEM: Asociacion Mexicana de Facultades y Escuelas de Medicina
AMMEF: Asociación Mexicana de Médicos en Formación
